# Long non‐coding RNA ADAMTS9‐AS1 suppresses colorectal cancer by inhibiting the Wnt/β‐catenin signalling pathway and is a potential diagnostic biomarker

**DOI:** 10.1111/jcmm.15713

**Published:** 2020-09-05

**Authors:** Ning Li, Juan Li, Qi Mi, Yan Xie, Peilong Li, Lili Wang, Helen Binang, Qing Wang, Yanlei Wang, Yingjie Chen, Yunshan Wang, Haiting Mao, Lutao Du, Chuanxin Wang

**Affiliations:** ^1^ Department of Clinical Laboratory The Second Hospital of Shandong University Jinan China; ^2^ Tumor Marker Detection Engineering Laboratory of Shandong Province Jinan China; ^3^ Department of Clinical Laboratory Qilu Hospital Jinan China; ^4^ Department of Clinical Laboratory Dezhou People's Hospital Dezhou China; ^5^ Department of General Surgery Qilu Hospital Jinan China; ^6^ The Laboratory Clinical Medical Research Center of Shandong Province Jinan China

**Keywords:** ADAMTS9‐AS1, colorectal cancer, EMT, exosomes, LncRNA

## Abstract

Long non‐coding RNAs (lncRNAs) have come out as critical molecular regulators of human tumorigenesis. In this study, we sought to identify and functionally characterize lncRNAs as potential mediators of colorectal cancer progression. We screened and identified a novel lncRNA, ADAMTS9‐AS1, which was significantly decreased in colorectal cancer tissues and was correlated with clinical outcome of patients according to The Cancer Genome Atlas (TCGA) database. In addition, ADAMTS9‐AS1 regulated cell proliferation and migration both in vitro and in vivo. Bioinformatics analysis revealed that overexpression of lncRNA‐ADAMTS9‐AS1 preferentially affected genes that were linked to proliferation and migration. Mechanistically, we found that ADAMTS9‐AS1 obviously suppressed β‐catenin, suggesting that Wnt signalling pathway participates in ADAMTS9‐AS1‐mediated gene transcriptional regulation in the suppression of colorectal tumorigenesis. Finally, we found that exosomal ADAMTS9‐AS1 could serve as a diagnostic biomarker for colorectal cancer with AUC = 0.835 and 95% confidence interval = 0.777‐0.911. Our data demonstrated that ADAMTS9‐AS1 might play important roles in colorectal cancer by suppressing oncogenesis. Targeting ADAMTS9‐AS1 may have potential clinical applications in colorectal cancer prognosis and treatment as an ideal therapeutic target. Finally, exosomal lncRNA‐ADAMTS9‐AS1 is a promising, novel diagnostic biomarker for colorectal cancer.

## 1 INTRODUCTION

Colorectal cancer (CRC) is the third most common cancer and the fourth most common cause of cancer deaths throughout the world.^1^, ^2^, ^3^ Despite the intensive investigations and the therapeutic improvements, over 50% of patients ultimately die from this disease due to late diagnosis and treatment,^4^ which makes early diagnosis a prerequisite for improving CRC patient's survival. However, presently, the methods available for CRC diagnosis, including carcinoembryonic antigen (CEA) and colonoscopy, are limited owing to their low sensitivity and specificity, high cost and discomfort to the patients.^5^, ^6^ This highlights the necessity to unravel other unknown mechanisms contributing to CRC malignancy and identify novel molecular diagnostic biomarkers.^7^

Long non‐coding RNAs (lncRNAs) are a class of limited protein‐coding potential transcripts with minimum lengths of 200 nucleotide bases.^8^, ^9^ LncRNAs play various regulatory functions in physiological and pathological processes, such as transcriptional interference, induction of chromatin remodelling and interaction with mRNAs, proteins and miRNAs.^10^, ^11^ Recent studies have shown that lncRNAs participate in various processes in carcinogenesis and progression including tumour proliferation and metastasis and conversely act as tumour suppressors depending on the tumour microenvironments.^12^, ^13^ However, the roles of lncRNAs in colorectal cancer are poorly understood.

The lncRNA‐ADAMTS9‐AS1 is an antisense lncRNA dysregulated in a number of tumours,^14^, ^15^, ^16^, ^17^ and dysregulated antisense RNAs have been implicated in the pathogenesis of various human malignancies.^18^, ^19^, ^20^, ^21^, ^22^, ^23^ However, its role in colorectal cancer has not been extensively studied.

Exosomes are small membrane‐bound extracellular vesicles with diameters of approximately 30‐150 nm. Virtually, all cells in organisms secrete extracellular vehicles (EVs) packed with DNA, RNA and proteins, including tumour cells,^24^ which play central roles in cell‐cell communication.^25^ Recent studies have suggested that exosomes secreted from tumour tissues or cells can be transferred to the circulation and the lncRNAs within exosomes could serve as candidate biomarkers in some tumours.^26^, ^27^, ^28^ In the present study, we investigated the involvement of ADAMTS9‐AS1 in transcriptional regulation, as well as its potential for application as a diagnostic marker for CRC. We provide evidence of the potential role of ADAMTS9‐AS1 in regulating colorectal tumorigenesis and the possibility of its application in CRC diagnosis and prognostic evaluation. Furthermore, we demonstrate that suppression of ADAMTS9‐AS1 is a potential clinical therapeutic strategy for CRC.

## 2 MATERIALS AND METHODS

### 2.1 Clinical patients tissues and serum sample cohorts

This study included multiple clinical cohorts with a total of 886 patients. These cohorts included patients from the publicly available TCGA data set (n = 698) and two clinical cohorts of CRC patients.

We obtained a total of 109 paired CRC and adjacent non‐tumour tissues from patients who were enrolled in Qilu Hospital of Shandong University, Jinan, China, from 2015 to 2017. All enrolled patients did not receive any preoperatnive therapies before sample collection and were characterized according to the eighth TNM staging of the American Joint Committee on Cancer system classification. All collected samples were snap‐frozen in liquid nitrogen and stored at −80°C. Clinical and pathological parameters of the clinical cohorts are provided in Table S1.

A total of 260 serum samples were used for our study, which included 130 CRC patients and 130 healthy volunteers. The samples were collected at the Qilu Hospital between March 2016 and August 2018. All the samples were collected 7 days before surgery or other therapies. At first, we detected the expression of ADAMTS9‐AS1 in the serum from 70 CRC patients and 70 healthy volunteers (training set), and we validated the diagnostic value of ADAMTS9‐AS1 in 60 CRC samples and 60 healthy controls (validation set). We isolated the serum by two‐step centrifugation protocol as follows: 2000 rpm for 10 min and then 12 000 rpm for 30 min at 4°C. Detailed clinical characteristics are listed in Table S3.

Our study was conducted in accordance with the Declaration of Helsinki and approved by the institutional review boards.

### 2.2 Public data set and gene expression signature identification

Identification of new therapeutic strategies for CRC is contingent upon identification of underexpressed or overexpressed molecules such as lncRNAs. To this end, the genome‐wide lncRNA expression profiles of primary tumours were investigated. The profiles which were of interest contained those lncRNAs that are lowly expressed in tumour tissues compared with normal tissues, and also those low‐expressed lncRNAs associated with poor prognosis from The Cancer Genome Atlas (TCGA). In the first step, analysis was carried out using RNA‐sequencing data taken from 698 tissue samples, in which 51 samples were normal colorectal tissues and the rest were colorectal cancer tissues. We performed a Wilcoxon signed‐rank test with the purpose of determining differential lncRNA expressions between normal and tumour groups. Additionally, to prioritize the potential candidates, we also calculated the log_2_ fold change and average expression levels as additional indicators. Using multivariate Cox regression analysis (n = 698), we assessed the prognostic performance of ADAMTS9‐AS1 based on survival analysis, in which the median value of the predictive risk score in TCGA data set was used as the cut‐off value.

### 2.3 Cell culture

All the cell lines including one human normal colonic epithelial cell line (CCD 841 CoN) and six human CRC cell lines (DLD‐1, SW480, HT29, HCT116, SW1116 and LOVO) were maintained in DMEM (Gibco) supplemented with 10% foetal bovine serum (FBS, Australia Origin, Gibco) in a 5% CO_2_ atmosphere at 37°C.

### 2.4 Overexpression or knockdown of ADAMTS9‐AS1 in colorectal cancer cells

SW116 and HT29 cells were plated at 5 × 10^6^ cells per well in 6‐well plates and transfected the ADAMTS9‐AS1 Lentivirus at MOI = 30 in Opti‐MEM supplemented with polybrene. Stable cell lines were selected for 5 days with 2 μg/mL puromycin. Three individual ADAMTS9‐AS18 shRNAs (shRNA ADAMTS9‐AS1 1#, 2# and 3#) and negative control shRNA vectors (Lnbio) were transfected into the CCD 841 CoN cell line, respectively, by Lipofectamine 2000 (Invitrogen), according to the manufacturer's instructions. All plasmids used were extracted using the Endo‐Free Plasmid Mini Kit (Omega Bio‐Tek), and the specific shRNA sequences used were as follows: shRNA1#: GGACTTGCAACTGTGACTTTC, shRNA2#: GGGAGCCCTGCAAAGCTAATC, shRNA3#: GGAATTCAAGCTTCTACAAGG.

### 2.5 RNA extraction and quantitative real‐time PCR (qRT‐PCR) analysis

TRIzol reagent (Thermo Fisher Scientific) was used to isolate total RNA from the tissues and then reversely transcribed into cDNA by TaKaRa Prime Script RT Reagent Kit (TaKaRa). After that, qRT‐PCR analyses were performed with SYBR Premix Ex Taq (TaKaRa) according to standard methodology. Relative expressions were calculated using the 2^−∆∆Ct^ method, by comparing expressions to those of GAPDH and U6. The specific primer sequences used were as follows: GAPDH, forward: 5′‐ACCCACTCCTCCACCTTTGAC‐3′, reverse: 5′‐TGTTGCTGTAGCCAAATTCGTT‐3′, ADAMTSA9‐AS1, forward: 5′‐CTAATCG CCAGGATTCCCTCC‐3′, reverse: 5′‐CCTGTTGTGGAGTTGCCCTT‐3′, cyclin D1, forward: 5′‐GATGCCAACCTCCTCAACGA‐3′, reverse: 5′‐GGAAGCGGTCCA GGTAGTTC‐3′, C‐Myc, forward: 5′‐CCCTCCACTCGGAAGGACTA‐3′, reverse: 5′‐GCTGGTGCATTTTCGGTTGT‐3′, β‐catenin, forward: 5′‐GGCTTGGAATGAG ACTGCTG, reverse: 5′‐GGTCCATACCCAAGGCATCC‐3′.

### 2.6 Western blot

All proteins were extracted with RIPA extraction reagent (Solarbio) supplemented with a protease inhibitor (Roche Applied Science). Phosphate‐buffered saline (PBS) supplemented with 5% bovine serum albumin and 1% Tween‐20 was used to block the polyvinylidene fluoride membranes (Merck‐Millipore) and then incubated with primary antibodies at 4°C overnight. The primary antibodies used were as follows: anti‐β‐actin (Zsbio, #TA‐09), anti‐E‐Cadherin (CST, #3195), anti‐N‐Cadherin (CST, #13116), anti‐vimentin (CST, #5741), anti‐Snail (CST, #3879), anti‐β‐Catenin (CST, #8480T), anti‐C‐Myc (CST, #5605), anti‐Cyclin D1 (CST, #2978), anti‐CD9 (CST, #13403) and anti‐TSG101 (Abcam, #ab125011). The signals were detected using Immobilon™ Western Chemiluminescent HRP Substrate.

### 2.7 Cell proliferation and colony formation assays

We used the xCELLigence RTCA DP instrument (ACEA Biosciences) to detect the cell proliferation which was shown by cell index in real time for 72 hours. We seeded 2000 stable cells (HT29, SW1116) per well in 6‐well plates and cultured them for approximately 2 weeks. After 15 minutes in 4% paraformaldehyde, the colonies were stained with 0.5% crystal violet (Solarbio) and counted.

### 2.8 5‐Ethynyl‐20‐deoxyuridine (EdU) assay

SW1116 and HT29 cells were seeded in 96‐well culture plates and cultured to 60%‐70% confluency. After this, they were transfected for 48 hours and then incubated for 2 hours before addition of 50 μmol/L EdU labelling media. According to the EdU assay kit (RiboBio), the DNA synthesis rates of CRC cell lines were characterized by the number of EdU‐positive cells counted in three random images per well by microscopy with 10× objective (Carl Zeiss).

### 2.9 Transwell assays and wound healing test

The transfected cancer cells were seeded in transwell chambers, with serum‐free media in the upper chambers and 20% serum added to the media in the lower chambers. After incubation at 37°C, 5% CO_2_ for 24 hours, the lower chambers were fixed with methanol and stained in 0.1% crystal violet. The cells were photographed and counted by microscopy with 10 × objective (Carl Zeiss). We also transferred 1 × 10^6^ cells per well to a 6‐well plate and used a pipette tip to make a wound after 24 hours. Meanwhile, we changed the medium to medium with 2% FBS and photographed the gap every 24 hours to show the migratory ability of the cells.

### 2.10 Immunofluorescence assay

Cells were seeded on coverslips and fixed with 4% paraformaldehyde. Twenty‐four hours (24 hours) later, goat serum (Solarbio) was used to block the cells and they were incubated with primary antibodies overnight at 4°C. After three washes with PBS, fluorescein‐conjugated goat anti‐rabbit secondary antibodies were added for 1 hour. At last, the cells were stained using 4, 6‐diamidino‐2‐phenylindole (DAPI, Beyotime, Shanghai, China) and photographed by microscopy (Carl Zeiss).

### 2.11 Exosome purification and identification

5 mL of serum samples was filtered through a 0.22‐μm pore polyvinylidene fluoride filter (Millipore). Subsequently, the serum was transferred to a fresh Beckman ultracentrifuge tube and PBS was added to make a total volume of 33.8 mL. This was then centrifuged for 120 minutes at 110 000 *g*, 4°C, using Optima™ XPN ultracentrifuge (Beckman Coulter) to extract the exosomes. Transmission electron microscopy (JEM‐1‐11 microscope, Japan) was used to photograph exosomes at 100 keV, and NanoSight NS300 instrument (Malvern Instruments Ltd. UK) equipped with NTA 3.0 analytical software (Malvern Instruments Ltd. UK) was used to determine the size distribution and concentration of exosomes.

### 2.12 Fluorescence in situ hybridization and subcellular fractionation (FISH)

The procedure was performed by using a FISH Hybridization Kit (GenePharma). Briefly, SW1116 and HT29 cells seeded on 48‐well culture plates were cultured to 60%‐70% confluency. After washing with PBS twice, the cells were incubated in 4% paraformaldehyde for 15 minutes. Fixed cells were treated with 0.1% buffer A for 15 minutes, washed with PBS twice and then incubated in 2 × buffer C for 30 minutes and subsequently dehydrated through 70%, 85% and 100% ethanol. The air‐dried cells were further incubated with 4 μmol/L fluorescence in situ hybridization (FISH) probe in buffer E at 37°C overnight. After washing by 0.1% buffer F, 2 × buffer C, the slide was stained with DAPI for detection.

### 2.13 Immunohistochemistry (IHC)

The tissue samples of tumour xenograft mice were fixed in 4% paraformaldehyde and embedded in paraffin for IHC staining. Briefly, after antigen retrieval and fixation, the antigens were blocked using goat serum. The tissue sections were then incubated at 4℃ with primary antibody overnight, the IgG‐HRP secondary antibody and DAB solution were used to stain, and the staining intensity was characterized by three grades: no staining, light yellow and yellow‐brown.

### 2.14 Animal experiments

This study was approved by the Committee on the Ethics of Animal Experiments of the Second Hospital of Shandong University. The stable cell lines HT29 were subcutaneously injected into the 4‐week‐old male BALB/c nude mice. The volumes of tumour were measured every 5 days and determined by *V* = 0.5 × *D *× *d* ^2^, where *D* is the longest diameter and d is the diameter perpendicular to the longest diameter.

### 2.15 Statistical analysis

Statistical analyses were performed using R 3.6.1, GraphPad Prism version 8.0, MedCalc version 12.3 programs and IBM SPSS version 20. The results were expressed as mean ± SD from at least three independent experiments, and the differences between two groups were analysed using Student's *t* test. The Mann‐Whitney *U* test or Kruskal‐Wallis test was used for evaluating the ADAMTS9‐AS1 expression levels and the pathologic difference among clinical cohort groups. ROC and AUC were used to determine the diagnostic value of ADAMTS9‐AS1. *P*‐values < .05 were considered as statistically significant.

## 3 RESULTS

### 3.1 Screening of differentially expressed lncRNAs related to CRC tumorigenesis and prognosis

**Figure 1:**
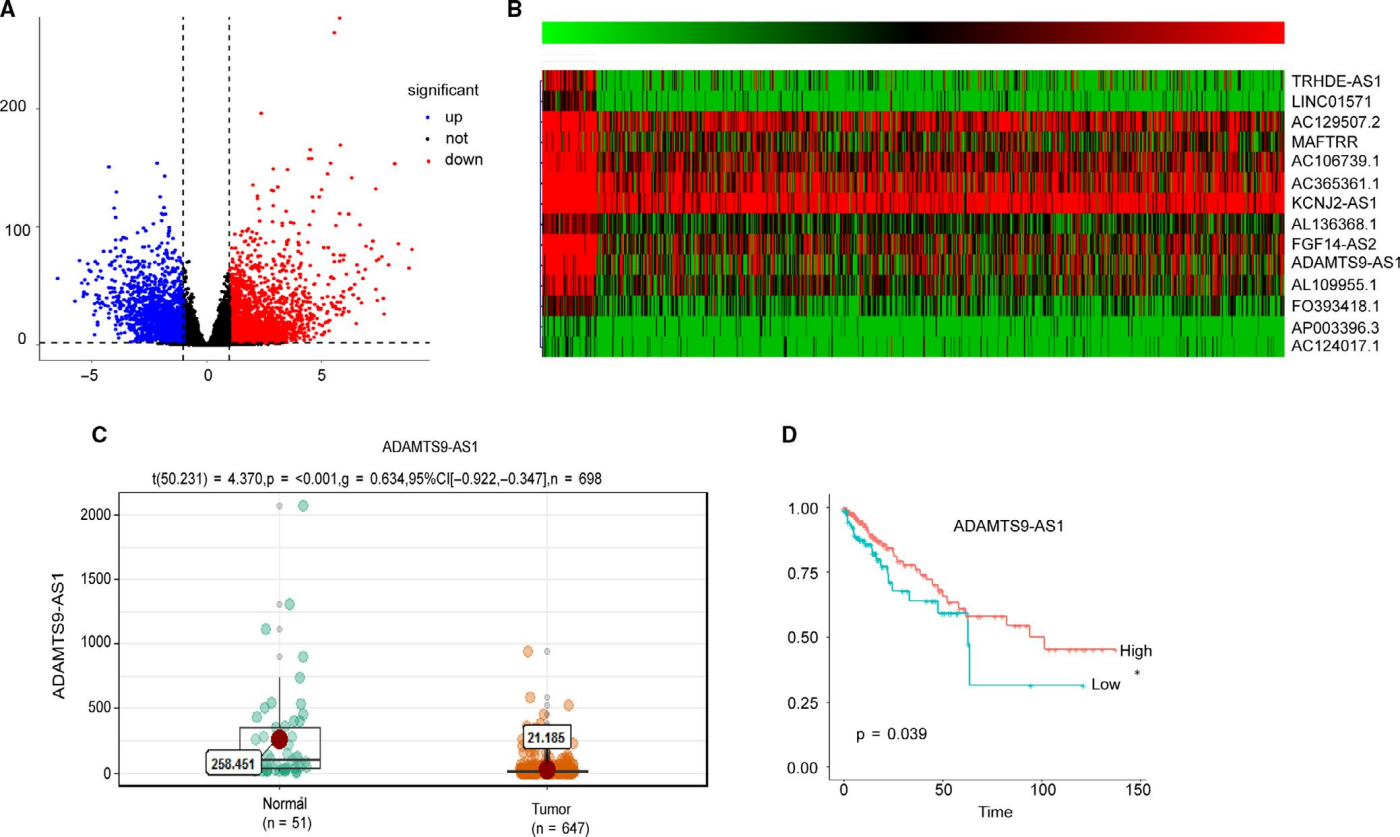
Screening of differentially expressed lncRNAs related to CRC tumorigenesis and prognosis. A, Relative expression of differentially expressed LncRNAs in CRC compared with normal tissues was analysed using TCGA database. B, Heat map of 14 candidate lncRNAs with top statistical significance. C, Relative expression of ADAMTS9‐AS1 in CRC compared with normal tissues was analysed using TCGA database. D, Kaplan‐Meier survival curve of ADAMTS9‐AS1

Based upon the study design illustrated in Figure 1A, we performed a genome‐wide, unbiased discovery to identify lncRNAs that play key roles in tumour suppression. In the TCGA discovery cohort, we first compared lncRNA expression profiles between normal and cancer groups and we identified the target lncRNAs with absolute log_2_ fold change difference of 1, a *P*‐value < .05 (Wilcoxon signed‐rank test) and an average expression level of greater than 10 transcripts per million. Based on Kaplan‐Meier curve and log‐rank test analysis using lncRNAs from the previous step, 14 candidate lncRNAs with top statistical significance (*P*‐value < .05) were further selected including ADAMTS9‐AS1, AL36536.1, KCNJ2‐AS1, FGF14‐AS2, AC129507.2, AC106739.1, MAFTRR, AL109955.1, TRHDE‐AS1, AL136368.1, FO393418.1, LINC01571, AP003396.3 and AC124017.1. Kruskal‐Wallis test analysis indicated that the 14 lncRNA expression levels in tumour tissues were significantly lower (all *P*‐values < .05) than those of adjacent normal tissues in TCGA data set (Figure 1B,C, Figure S1). Moreover, according to the Kaplan‐Meier analysis, patients with low expression levels of the 14 lncRNAs experienced significantly poorer prognosis (all *P*‐values < .05) than those with high expression levels (Figure 1D, Figure S2).

### 3.2 ADAMTS9‐AS1 is down‐regulated in colorectal cancer tissues and cell lines

**Figure 2:**
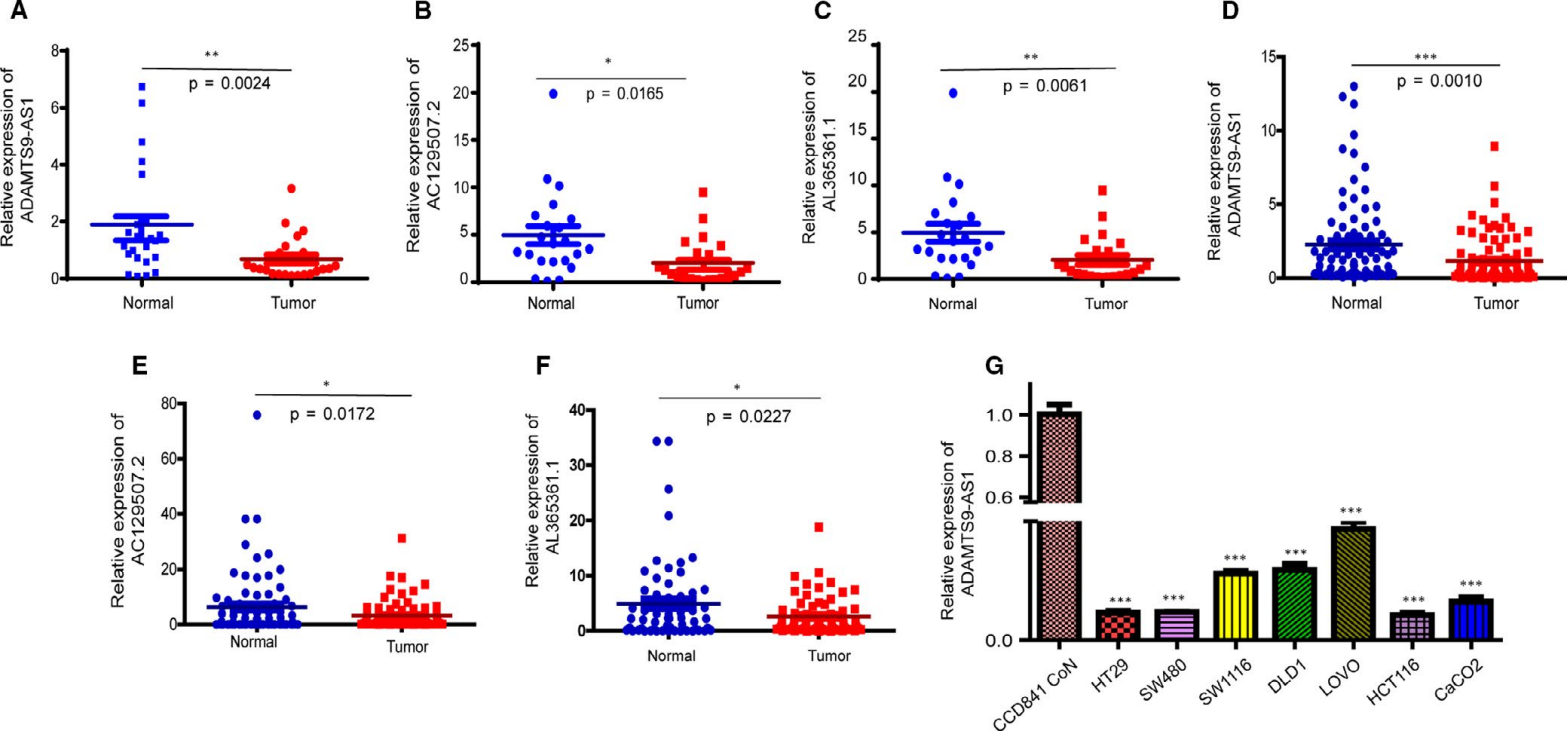
ADAMTS9‐AS1 is down‐regulated in colorectal cancer tissues and cell lines. A, D, qPCR analysis of ADAMTS9‐AS1 expression in CRC and matched adjacent normal tissues. B, E, qPCR analysis of AC129507.2 expression in CRC and matched adjacent normal tissues. C, F, qPCR analysis of AL365361.1 expression in CRC and matched adjacent normal tissues. G, ADAMTS9‐AS1 expression was measured in normal colon epithelial cell line (CCD 841 CoN) and established CRC cell lines (DLD‐1, SW480, HT29, HCT116, SW1116 and LOVO) using qPCR. The data are presented as the means ± SD from three biological replicates. **P < .01, ***P < .001

In the training phase, 24 paired CRC and matched adjacent normal tissues in cohort 1 were used to validate the relative expression of these 14 selected lncRNAs by RT‐qPCR. Paired *t* test analysis indicated that ADAMTS9‐AS1 (*P* = .0024), AC129507.2 (*P* = .0165) and AL365361.1 (*P* = .0061) were down‐regulated in CRC tissues compared with adjacent normal tissues (Figure 2A‐C). We further validated the expression levels and efficiencies of these three lncRNAs estimated, in 85 paired CRC and matched adjacent normal tissues by employing another independent validation set. The distributions of age, gender and other clinical characteristics between CRC and control samples in the training and validation sets were not significantly different (Table S2). However, ADAMTS9‐AS1 was significantly decreased in CRC tissues compared with adjacent normal tissues (Figure 2D‐F). Due to our observation of substantial down‐regulation of ADAMTS9‐AS1 in tumour tissues compared to adjacent normal tissues, RT‐qPCR was used to verify ADAMTS9‐AS1 expression levels in CRC cell lines normalized to GAPDH. All seven colorectal cancer cell lines (DLD‐1, SW480, HT29, HCT116, SW1116 and LOVO) expressed lower ADAMTS9‐AS1 than the normal CRC cell line, CCD 841 CoN (Figure 2E).

### 3.3 Overexpression of ADAMTS9‐AS1 suppresses tumour growth in vitro and in vivo

**Figure 3:**
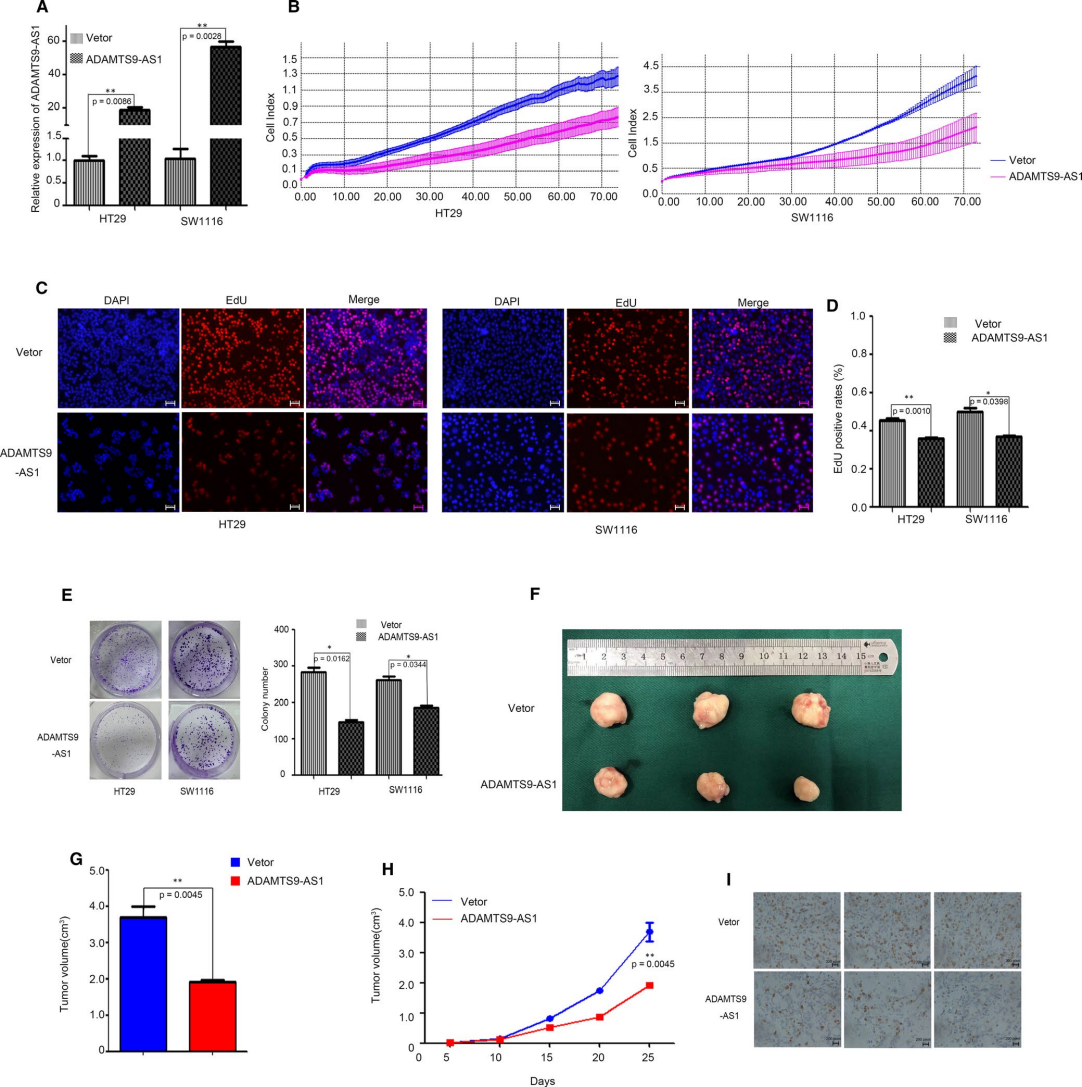
Overexpression of ADAMTS9‐AS1 suppresses tumour growth in vitro and in vivo. A, qPCR analysis of ADAMTS9‐AS1 expression in Vector‐transfected and Lentivirus‐transfected CRC cells. B, xCELLigence system was used to monitor the cell growth dynamics of Vector‐transfected and Lentivirus‐transfected CRC cells. C, D, EdU staining assays were performed to detect the cell cycle. E, Colony formation assay was performed to determine the proliferation of CRC cells. F‐H, The stable ADAMTS9‐AS1‐overexpressing cell line, HT29, was used for in vivo assays. The tumours from two groups of nude mice are shown, and tumour growth curves were measured and shown after the injection. The tumour volume was calculated every 5 d. I, Ki67 protein levels in tumour tissues from oe‐ADAMTS9‐AS1 or negative control HT29 cells were determined by immunohistochemistry. The data represent the mean ± SD from three independent experiments. *P < .05, **P < .01

To investigate the biological functions of ADAMTS9‐AS1 in the tumorigenesis and development of CRC, we transfected the ADAMTS9‐AS1 Lentivirus to overexpress ADAMTS9‐AS1 in two CRC cell lines, SW1116 and HT29 (Figure 3A). We examined the cell growth using the cell growth dynamics (monitored using the xCELLigence system), and the results showed that ADAMTS9‐AS1 significantly inhibited CRC cell proliferation (Figure 3B). In addition, colony formation assay showed that the number of colonies of CRC cells was significantly reduced after ADAMTS9‐AS1 overexpression (Figure 3E). Furthermore, we investigated the cell cycle distribution to ascertain the molecular mechanism by which ADAMTS9‐AS1 suppresses cell proliferation. The number of EdU‐positive cells was decreased after overexpression of ADAMTS9‐AS1, indicating cell cycle inhibition (Figure 3C,D). To determine the tumour suppressor functions in vivo, HT29 cells were injected into male nude mice after stably transfecting ADAMTS9‐AS1 and control Lentivirus. We measured the longest diameter and the diameter perpendicular to the longest diameter every 5 days. Obviously, the average tumour sizes were significantly smaller after overexpression of ADAMTS9‐AS1 and the growth rates of tumours also indicated that ADAMTS9‐AS1 could inhibit tumour growth in vivo (Figure 3F‐H). In addition, immunohistochemical staining for Ki‐67, which is the cell proliferation marker, showed a lower expression in ADAMTS9‐AS1‐overexpressing tumours (Figure 3I). Collectively, these results confirmed the in vitro and in vivo tumour suppressor activity of ADAMTS9‐AS1 in CRC.

### 3.4 Overexpression of ADAMTS9‐AS1 suppresses tumour metastasis and epithelial‐mesenchymal transition (EMT) in vitro

**Figure 4:**
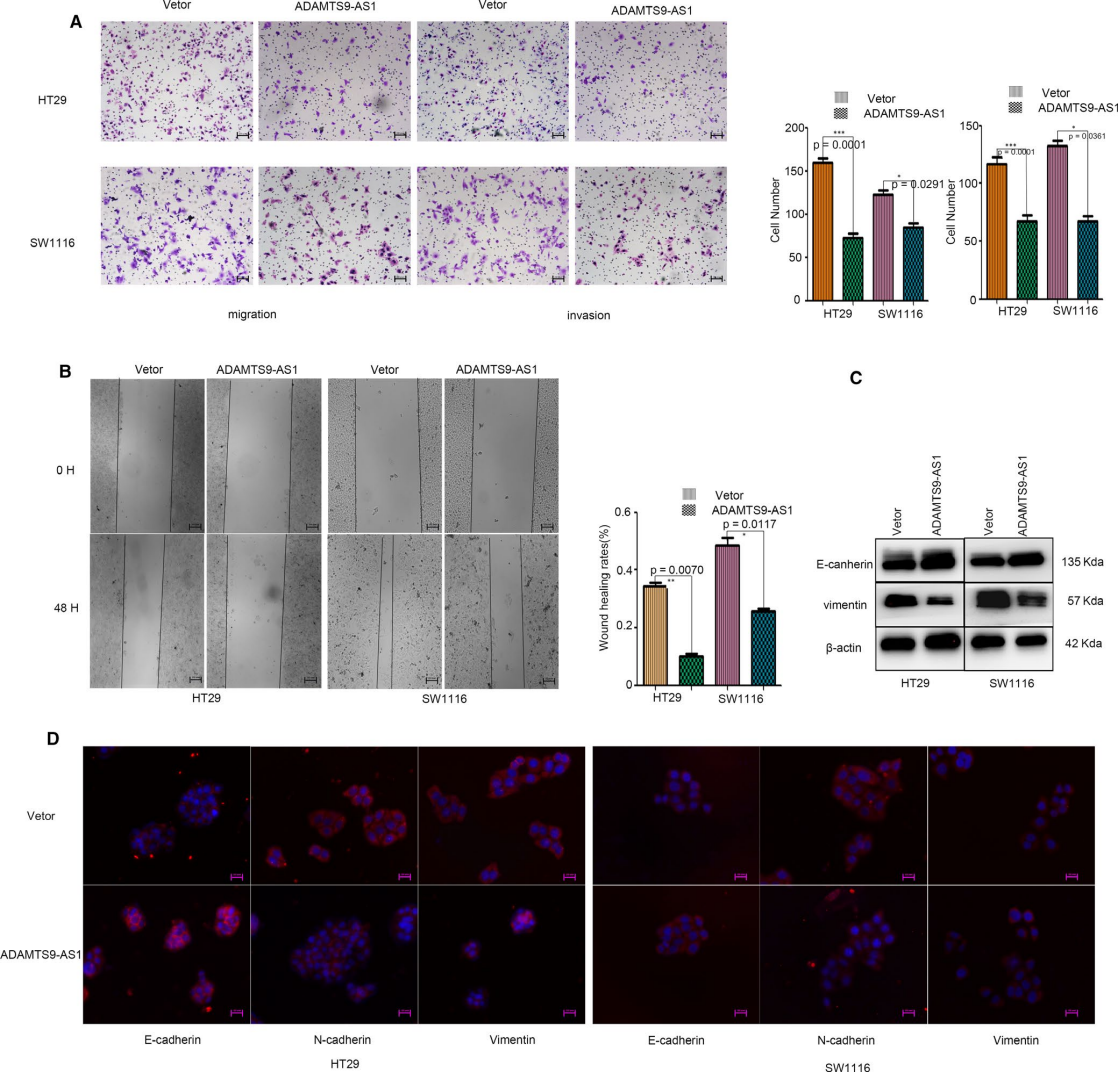
Overexpression of ADAMTS9‐AS1 suppresses tumour metastasis and epithelial‐mesenchymal transition (EMT) in vitro. A, B, Transwell assays and the wound healing test were used to detect the migratory and invasive abilities of CRC cells. C, D, The epithelial‐mesenchymal transition (EMT) markers were detected by Western blot and immunofluorescence. **P < .01, ***P < .001, ****P < .0001

We also investigated whether ADAMTS9‐AS1 influences the migration of CRC cells. Transwell and wound healing assays showed that ADAMTS9‐AS1 overexpression significantly impaired the migratory and invasive ability of CRC cells (Figure 4A,B). As determined by Western blot and immunofluorescence assays, ADAMTS9‐AS1 overexpression significantly increased the levels of E‐cadherin but decreased those of N‐cadherin, and vimentin in SW1116 and HT29 cells (Figure 4C,D), indicating that epithelial‐mesenchymal transition (EMT) was obviously suppressed.

### 3.5 ADAMTS9‐AS1 is preferentially localized to the nucleus and affects multiple cancer‐related pathways involved in cell proliferation and metastasis

**Figure 5:**
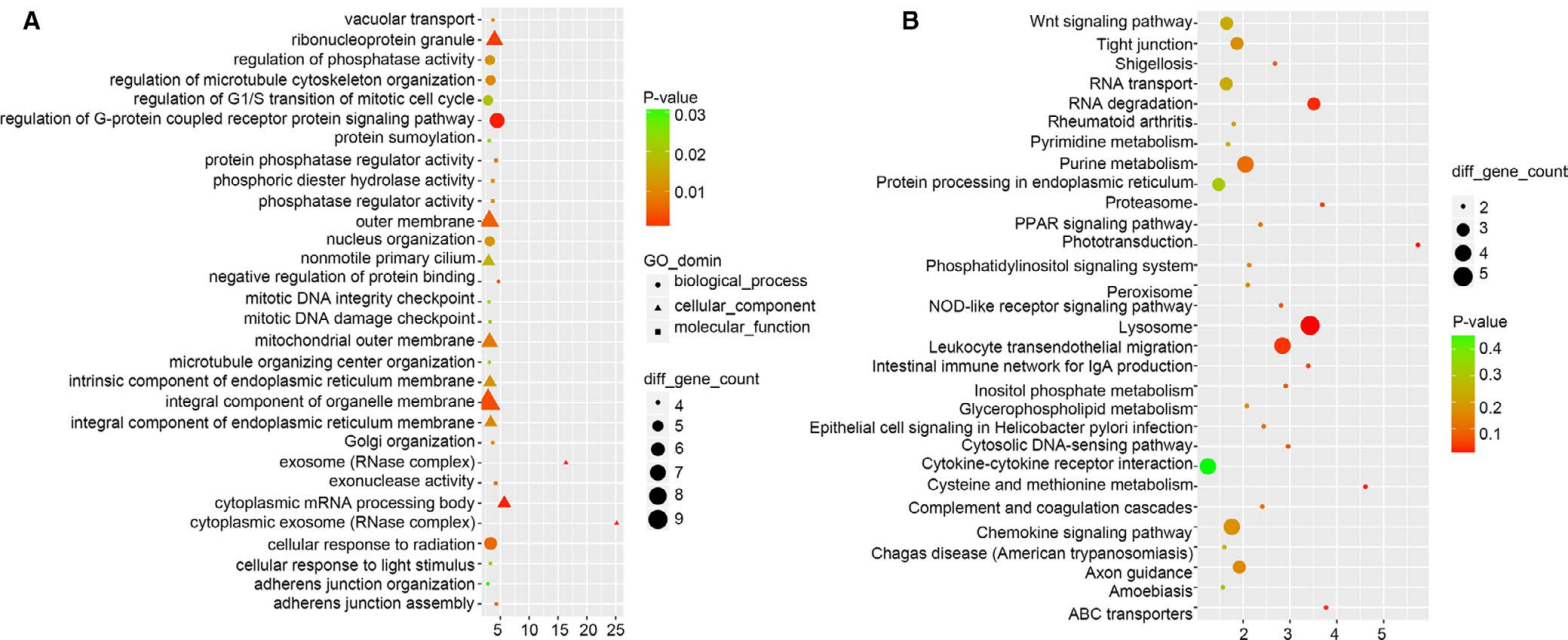
ADAMTS9‐AS1 is preferentially localized to the nucleus and affects multiple cancer‐related pathways involved in cell proliferation and metastasis. A, The GO terms for biological process, cellular component and molecular function of lncRNA ADAMTS9‐AS1. B, Kyoto Encyclopedia of Genes and Genomes (KEGG) pathway analysis of lncRNA ADAMTS9‐AS1

To reveal the potential biological functions of ADAMTS9‐AS1, we conducted Gene Ontology (GO) and Kyoto Encyclopedia of Genes and Genomes (KEGG) pathway analysis. The most significant GO terms for biological process, cellular component and molecular function indicated that ADAMTS9‐AS1 is mainly involved in the regulation of cell cycle, activation of pathways and protein phosphatase regulator activities (Figure 5A). Similarly, KEGG pathway analysis showed enrichments in Wnt signalling pathway, RNA transport, PPAR signalling pathway and lysosome (Figure 5B). In addition, analysis of the cellular localization of ADAMTS9‐AS1 by FISH and qPCR unveiled that ADAMTS9‐AS1 is preferentially localized to the nucleus (Figure S3A,B). Figure S3C shows the predicted target genes of ADAMTS9‐AS1.

### 3.6 ADAMTS9‐AS1 negatively regulated the Wnt signalling pathway

**Figure 6:**
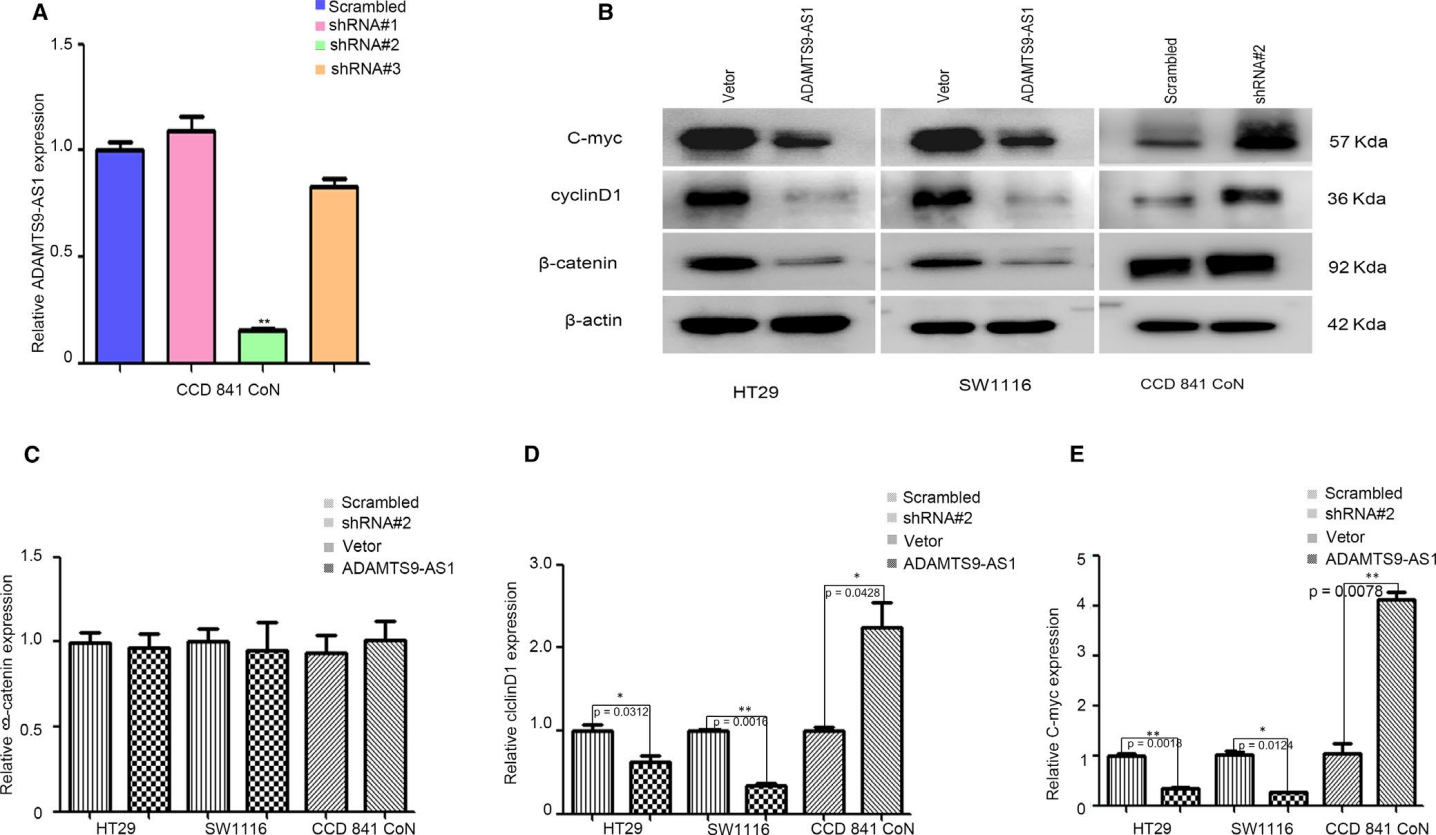
ADAMTS9‐AS1 negatively regulated the Wnt signalling pathway. A, qPCR analysis of ADAMTS9‐AS1 expression in control, shRNA 1#, shRNA 2# and shRNA 3# in CCD 841 CoN cells. B‐E, mRNA and protein levels of β‐catenin and the downstream targets of C‐Myc and cyclin D1. All experiments were repeated at least three times, and representative data are shown. Data are presented as mean ± SEM. **P < .01, ***P < .001, ****P < .0001

According to the KEGG pathway analysis, we identified that Wnt signalling pathway may be a potential target of ADAMTS9‐AS1. Thus, we investigated β‐catenin, which plays a crucial role in Wnt signalling pathway, by RT‐qPCR and Western blot, when ADAMTS9‐AS1 was overexpressed or inhibited in SW1116, HT29 and CCD 841 CoN cells. As shown in Figure 6A, the knockdown effect was best using shRNA2# compared with shRNA1# and shRNA3#. Thus, shRNA2# was used for subsequent experiments. The protein levels of β‐catenin were significantly decreased or increased by ADAMTS9‐AS1 overexpression or inhibition, respectively (Figure 6B), whereas the mRNA levels remained unchanged (Figure 6C). Meanwhile, the mRNA and protein levels of C‐Myc and cyclin D1—the Wnt/β‐catenin downstream genes—were decreased in ADAMTS9‐AS1‐transduced cells but were increased in ADAMTS9‐AS1‐silenced cells (Figure 6B,D,E). Collectively, these results suggest that ADAMTS9‐AS1 suppresses the Wnt/β‐catenin signalling pathway.

### 3.7 Exosomal ADAMTS9‐AS1 may serve as a diagnostic marker for CRC

**Figure 7:**
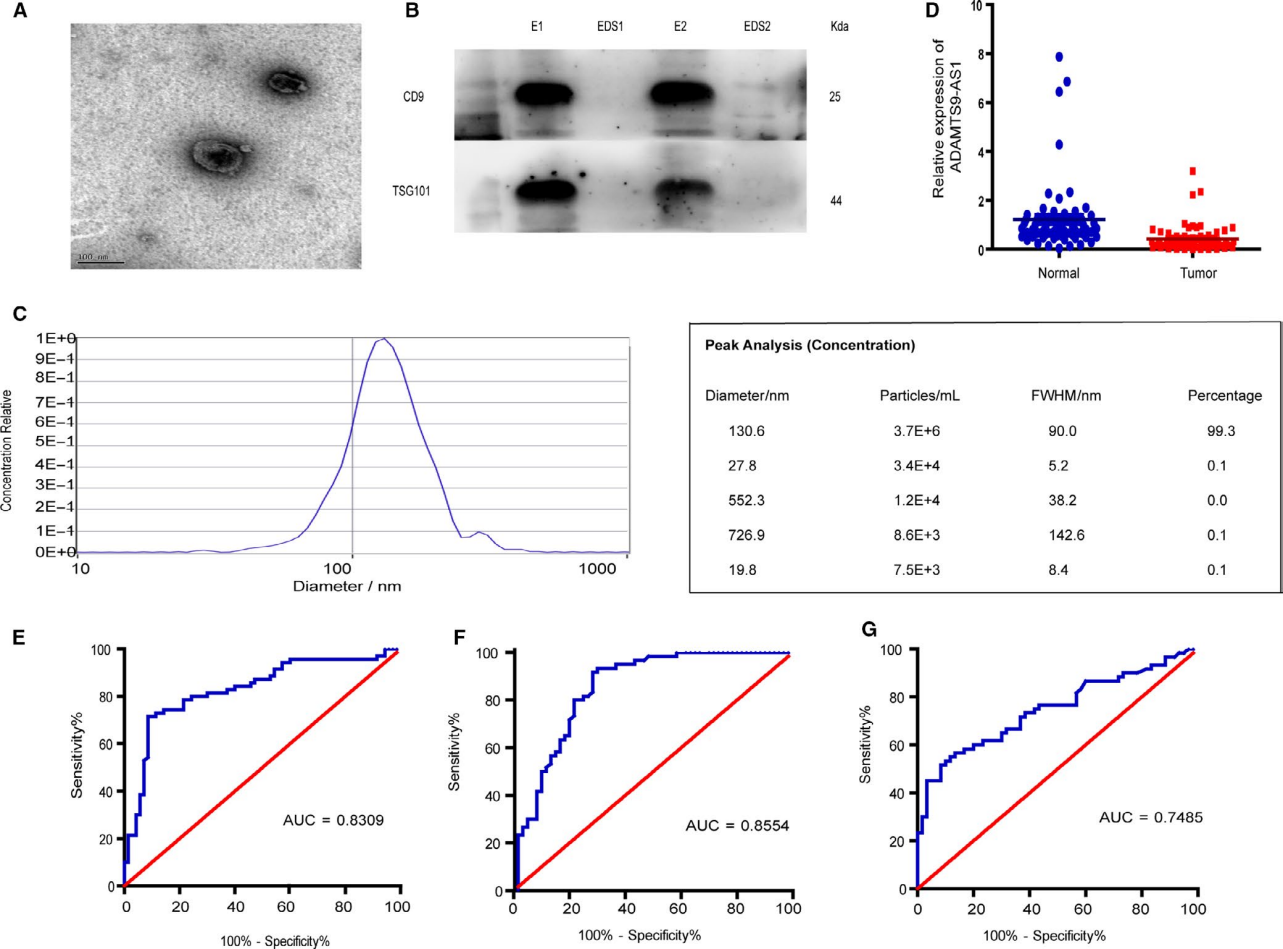
Exosomal ADAMTS9‐AS1 may serve as a diagnostic marker for CRC. A, Representative TEM images of serum exosomes, scale bar, 100 nm. B, Western blot analysis of TSG101 and CD9 in exosomes and exosome‐depleted supernatants (EDS). C, NTA of the size distribution and number of exosomes. D, RNA expression of exosomal ADAMTS9‐AS1 was measured in the serum of healthy controls (n = 130) and the serum of CRC patients (n = 130) using qPCR. E, F, ROC curve for serum exosomal ADAMTS9‐AS1 for the discrimination of patients with CRC from normal healthy individuals both in training set and in validation set. **P < .01, ***P < .001, ****P < .0001

After extraction of exosomes from serum, we used the transmission electron microscopy (TEM) and nanoparticle tracking analysis (NTA) to ensure the exosomes were successfully isolated. The TEM showed that exosomes have diameters of 60‐150 nm with typical cup‐shaped, round morphologies (Figure 7A), and NTA showed that the sizes of exosomes were mainly 130.6 nm in diameter (Figure 7C). In addition, Western blot analysis demonstrated higher concentrations of exosome protein markers CD9 and TSG101 (Figure 7B). These results suggested that the exosomes were successfully isolated from the serum.

We then sought to explore the diagnostic value of serum exosomal ADAMTS9‐AS1 in 260 serum samples. In the training phase, we found that compared with healthy controls, serum exosomal ADAMTS9‐AS1 expression levels were markedly reduced in CRC patients (Figure 7D). Moreover, ROC curve analyses showed that exosomal ADAMTS9‐AS1 had a strong capability of distinguishing CRC patients from healthy controls with AUC value of 0.831, 95% CI: 0.758‐0.889, and the sensitivity and specificity of 91.4% and 71.4%, respectively (Figure 7E). To further verify the diagnostic performance of ADAMTS9‐AS1, we detected ADAMTS9‐AS1 expression levels in the validation set, the results showed that the AUC of ADAMTS9‐AS1 was 0.853, with a sensitivity of 71.7% and a specificity of 91.7% (95% CI = 0.777‐0.911) (Figure 7F), and the AUC of CEA in this set was 0.748, with a sensitivity 56.7% of and a specificity of 86.7% (95% CI = 0.660‐0.838), which suggested that exosomal ADAMTS9‐AS1 is a promising serum biomarker for the diagnosis of CRC.

## 4 DISCUSSION

Numerous studies have demonstrated lncRNAs as important regulatory molecules, both in tumour suppressor and oncogenic pathways, whose mechanisms have advanced the understanding of human cancers.^29^ In recent years, the involvement of lncRNAs in human malignancies has attracted enormous attention.^30^ Several lncRNAs have been identified to be aberrantly expressed and involved in regulating multiple processes such as tumour growth and metastasis in CRC.^31^, ^32^ However, the mechanisms by which lncRNAs interact with proteins to directly regulate signalling are still largely underexplored.

In the present study, we screened TCGA database and identified a novel CRC‐associated lncRNA, ADAMTS9‐AS1, which has an obviously decreased expression level in CRC compared with normal colorectal tissues. We also verified ADAMTS9‐AS1 expression levels in CRC clinical samples, and the Kaplan‐Meier analysis showed that low expression level of ADAMTS9‐AS1 correlated with significantly poorer prognosis than those with high expression levels. Previous evidence has reported down‐regulation of ADAMTS9‐AS1 in ovarian cancer, colon adenocarcinoma and breast cancer,^14^, ^15^, ^16^, ^17^ further validating our results, and a number of documented studies have also recorded the participation of down‐regulated lncRNAs in pathological processes.^33^, ^34^, ^35^ The correlation of ADAMTS9‐AS1 with poor prognosis in our study may imply that ADAMTS9‐AS1 is a possible marker of unfavourable outcome for colorectal cancer patients. Interestingly, several preceding studies have also implicated dysregulated lncRNA expressions with the survival probabilities of patients.^36^, ^37^, ^38^, ^39^ Our findings suggest that ADAMTS9‐AS1 plays an important role during CRC tumorigenesis.

Tumour formation is caused by continuous cell proliferation and limited cell differentiation, but the molecular mechanism of the Wnt/β‐catenin pathway in colorectal cancer is still unclear. Related molecular biology experiments in our study demonstrated that overexpression of ADAMTS9‐AS1 suppressed cell proliferation and migration, by inhibiting the Wnt signalling pathway to impede cell proliferation and migration in CRC.

Abnormally activated Wnt pathways are closely related to a variety of human diseases, including malignancies.^39^, ^40^ The Wnt pathway is involved in the development and progression of colorectal cancer by affecting life activities such as cell growth, proliferation and differentiation, which are the hallmark of colorectal carcinogenesis.^41^, ^42^ Previous studies have shown that intestinal formation requires activation of the Wnt signalling pathway. Excessive activation of this pathway leads to intestinal lesions, and approximately 90% of patients with colorectal cancer have abnormalities in the Wnt signalling pathway.^43^ It has been established that abnormal activation of the Wnt pathway is the main cause of the development and progression of colorectal cancer.^44^, ^45^ In other words, our study provides additional evidence of the role of lncRNAs in regulating pathologic processes of cancers and deepens the current understanding of the participation of lncRNAs in biological regulation.^46^, ^47^, ^48^ What's more, overexpression of ADAMTS9‐AS1 could down‐regulate β‐catenin and correspondingly decrease the downstream genes C‐Myc and cyclin D1, which is the same as E‐cadherin. On the other hand, knockdown of ADAMTS9‐AS1 could up‐regulate β‐catenin and increase the expression of C‐Myc and cyclin D1 and also promote the EMT process. These findings demonstrate that ADAMTS9‐AS1 suppresses tumour proliferation, invasion and migration mainly by inhibiting activation of the Wnt/β‐catenin signalling pathway.

Furthermore, to investigate the regulatory mechanism involved, we explored the cellular localization of ADAMTS9‐AS1 by FISH and qPCR. These unveiled that ADAMTS9‐AS1 is preferentially localized to the nucleus. In addition, we predicted the target genes by lncRNA trans and cis prediction. The results showed the potential target genes in the pathway and provide insight into the various components of the Wnt/β‐catenin signalling pathway and the mechanisms of interaction between them. These could be a pointer to effective antagonists for the development of specific targets of the Wnt/β‐catenin signalling pathway required for prevention and early treatment of colorectal cancer.

Identification of early non‐invasive markers for diagnosis is a major step in ameliorating mortality due to cancers, because late diagnosis is the major cause of the high death rates associated with human cancers.^49^ The results of our exosomal analysis of ADAMTS9‐AS1 expression suggest the potential role of this lncRNA as a diagnostic marker for CRC. This is corroborated with the findings of numerous previous studies suggesting the application of liquid biopsies in general, and exosomal lncRNAs in particular, in the diagnosis of malignant disorders^27^, ^50^, ^51^

The present study found down‐regulated expression of ADAMTS9‐AS1 in CRC tissues negatively correlated with the prognosis of patients. We demonstrated that ADAMTS9‐AS1 suppresses colorectal tumorigenesis both in vitro and in vivo by inhibiting the Wnt/β‐catenin signalling pathway. We also unveiled that serum exosomal ADAMTS9‐AS1 is a potential diagnostic marker with a strong capability of distinguishing CRC patients from healthy controls.

In conclusion, the present study provides evidence of the potential role of ADAMTS9‐AS1 in regulating colorectal tumorigenesis and the possibility of its application as a diagnostic and prognostic marker for CRC. We also revealed that suppression of ADAMTS9‐AS1 is a potential clinical therapeutic strategy for colorectal cancer. However, further studies are required to conclusively validate the potential clinical application of ADAMTS9‐AS1 in the management and treatment of CRC.

## CONFLICT OF INTEREST

The authors declare that they have no conflict of interest.

## AUTHOR CONTRIBUTIONS

**Chuanxin Wang:** Conceptualization (lead); funding acquisition (lead); project administration (lead). **Lutao Du:** Methodology (equal); project administration (equal); supervision (equal). **Ning Li:** Conceptualization (supporting); data curation (lead); formal analysis (lead); investigation (lead); methodology (lead); validation (lead); visualization (lead); writing‐original draft (lead); writing‐review and editing (lead). **Juan Li:** project administration (supporting); supervision (equal); writing‐original draft (equal); writing‐review and editing (equal). **Qi Mi:** Data curation (equal); visualization (equal). **Yan Xie:** Software (equal); validation (equal). **Pei Long Li:** Supervision (supporting). **Li Li Wang:** Resources (equal); supervision (equal). **Helen Binang:** Writing‐review and editing (equal). **Qing Wang:** Resources (supporting). **Lei Yan Wang:** Resources (lead). **Ying Jie Chen:** Validation (equal). **Yunshan Wang:** Conceptualization (supporting); supervision (equal). **Hai Ting Mao:** Supervision (supporting).

## Supporting information

Figure S1

Figure S2

Figure S3

Table S1

Table S2

Table S3

## Data Availability

The data used to support the findings of this study are available from the corresponding author upon reasonable request.
