# Supplementary figures and images for: Long non‐coding RNA ADAMTS9‐AS1 suppresses colorectal cancer by inhibiting the Wnt/β‐catenin signalling pathway and is a potential diagnostic biomarker
Source: J Cell Mol Med. 2020 Sep 5;24(19):11318–29. doi: 10.1111/jcmm.15713 (PMC7576284; doi:10.1111/jcmm.15713)

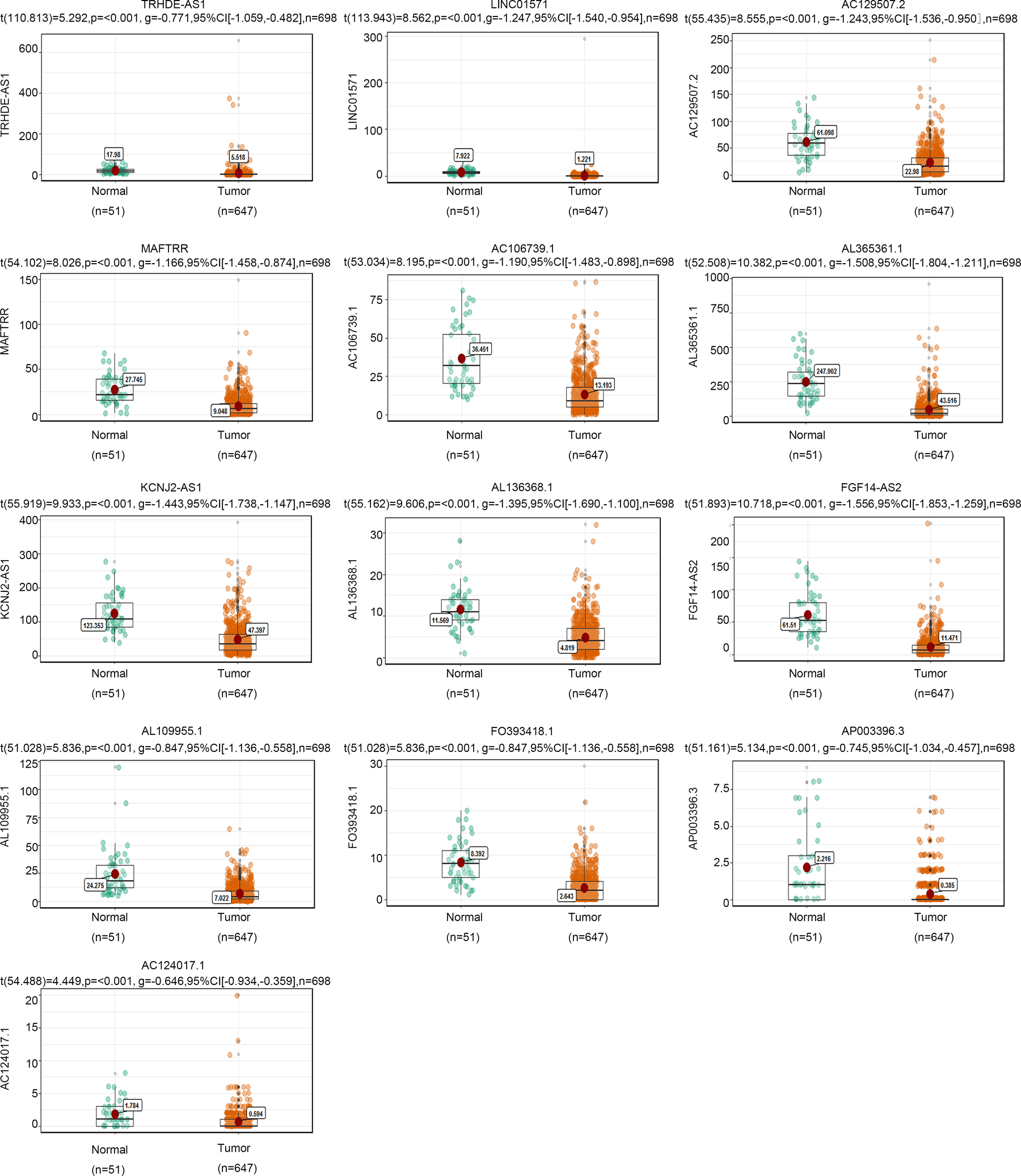

Supplement: Supplementary file 1 — Figure S1 [file JCMM-24-11318-s001.tif]

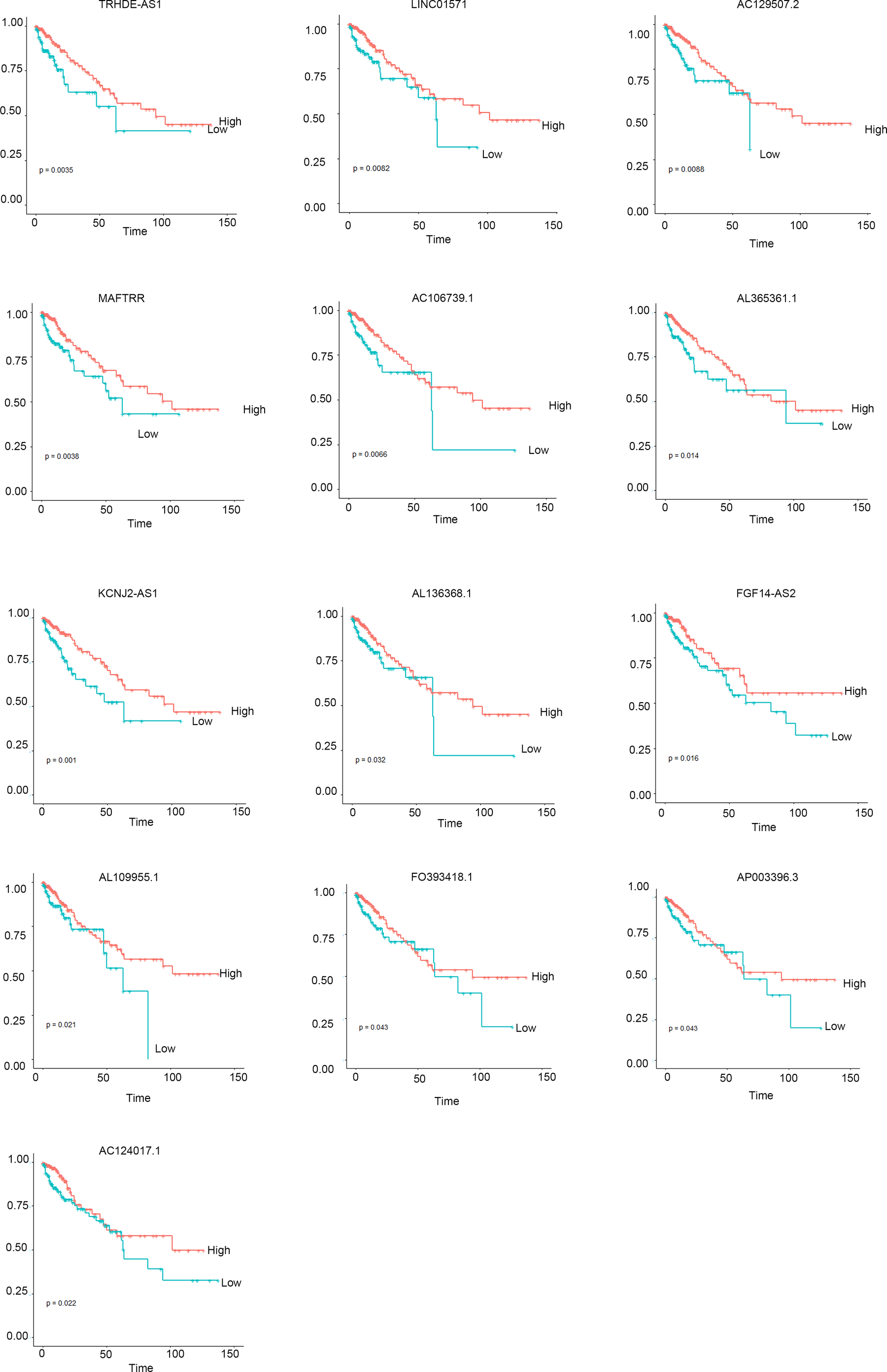

Supplement: Supplementary file 2 — Figure S2 [file JCMM-24-11318-s002.tif]

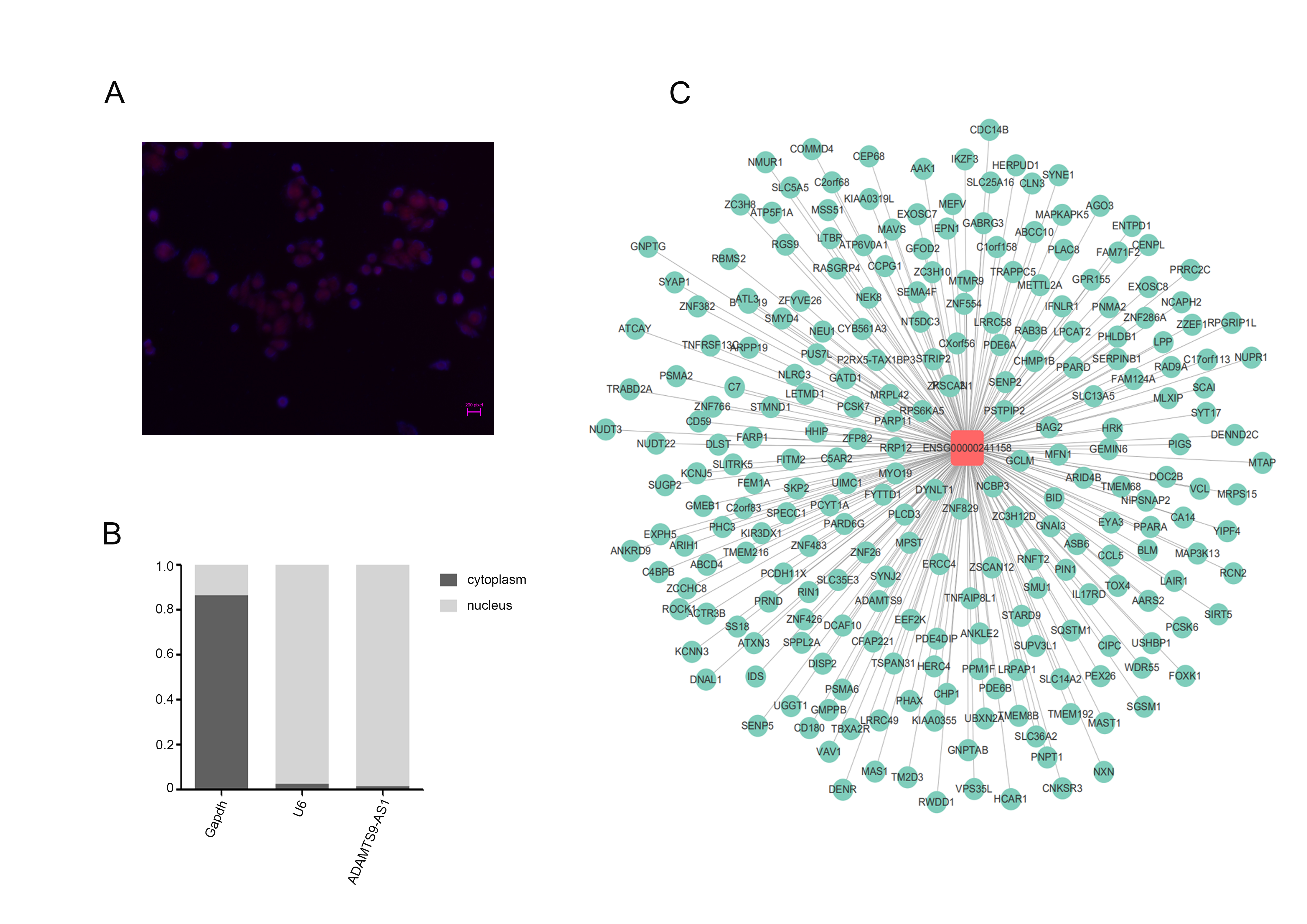

Supplement: Supplementary file 3 — Figure S3 [file JCMM-24-11318-s003.tif]
