# Supplementary material for: Long non‐coding RNA ADAMTS9‐AS1 suppresses colorectal cancer by inhibiting the Wnt/β‐catenin signalling pathway and is a potential diagnostic biomarker
Source: J Cell Mol Med. 2020 Sep 5;24(19):11318–29. doi: 10.1111/jcmm.15713 (PMC7576284; doi:10.1111/jcmm.15713)
Supplement: Supplementary file 4 — Table S1 [file JCMM-24-11318-s004.docx]

**Table S1. Clinic-pathological characteristics of patients and demographic information of controls enrolled in the study.**

| **Variable** | **Training set** | **Validation set** | **P-Value** |
| --- | --- | --- | --- |
|  | **n=24** | **n=85** |  |
| **CRC Patients (number)** | 24 | 85 |  |
| **Age (years)** |  |  | **0.6397** |
| ≤62 | 14 (58.33%) | 40(47.05%) |  |
| >62 | 10 (41.67%) | 45 (52.95%) |  |
| **Sex** |  |  | **0.4875** |
| Male | 16 (55.71%) | 50 (58.33%) |  |
| Female | 8 (44.29%) | 35 (41.67%) |  |
| **Tumor location** |  |  | **0.4584** |
| Rectum | 18(75.00%) | 57(67.05%) |  |
| colon | 6(25.00%) | 28(32.95%) |  |
| **Tumor stage** |  |  | **0.7327** |
| Ta-T2 | 3 (12.50%) | 13 (15.29%) |  |
| T3-T4 | 21 (87.50%) | 72 (84.71%) |  |
| **Lymph node metastasis** |  |  | **0.6808** |
| Negative | 11 (45.83%) | 43 (50.58%) |  |
| Positive | 13 (54.17%) | 42 (49.42%) |  |
| **Distant metastasis** |  |  | **0.1688** |
| No | 22(91.67%) | 83(97.64%) |  |
| Yes | 2(8.33%) | 2(2.36%) |  |
