# Supplementary material for: Long non‐coding RNA ADAMTS9‐AS1 suppresses colorectal cancer by inhibiting the Wnt/β‐catenin signalling pathway and is a potential diagnostic biomarker
Source: J Cell Mol Med. 2020 Sep 5;24(19):11318–29. doi: 10.1111/jcmm.15713 (PMC7576284; doi:10.1111/jcmm.15713)
Supplement: Supplementary file 5 — Table S2 [file JCMM-24-11318-s005.docx]

**Table S2. Demographic and clinical characteristics of individuals with CRC who**

**provide tissues**

| **Sample ID** | **Gende** | **Age** | **T** | **N** | **M** | **Tumor** |
| --- | --- | --- | --- | --- | --- | --- |
|  |  | **Year** | **Stage** | **Stage** | **Stage** | **Location** |
| SHCRC-T-1 | Male | 79 | 3 | 0 | 0 | colon |
| SHCRC-T-2 | Female | 32 | 4a | 1b | 0 | rectum |
| SHCRC-T-3 | Male | 68 | 4a | 0 | 0 | rectum |
| SHCRC-T-4 | Male | 77 | 4a | 1a | 0 | colon |
| SHCRC-T-5 | Male | 50 | 2 | 0 | 0 | rectum |
| SHCRC-T-6 | Male | 27 | 4a | 0 | 0 | rectum |
| SHCRC-T-7 | Female | 51 | 4a | 2b | 1b | rectum |
| SHCRC-T-8 | Female | 56 | 4a | 1a | 0 | colon |
| SHCRC-T-9 | Female | 54 | 4a | 1a | 0 | colon |
| SHCRC-T-10 | Female | 87 | 2 | 0 | 0 | rectum |
| SHCRC-T-11 | Female | 56 | 4a | 2a | 0 | rectum |
| SHCRC-T-12 | Male | 59 | 2 | 0 | 0 | rectum |
| SHCRC-T-13 | Male | 69 | 3 | 2a | 0 | rectum |
| SHCRC-T-14 | Male | 62 | 4a | 2a | 0 | rectum |
| SHCRC-T-15 | Male | 60 | 4a | 0 | 0 | colon |
| SHCRC-T-16 | Male | 82 | 4a | 0 | 0 | rectum |
| SHCRC-T-17 | Female | 49 | 4a | 0 | 0 | colon |
| SHCRC-T-18 | Male | 68 | 4a | 1a | 0 | rectum |
| SHCRC-T-19 | Male | 57 | 4a | 1a | 0 | rectum |
| SHCRC-T-20 | Male | 40 | 4b | 0 | 0 | rectum |
| SHCRC-T-21 | Male | 76 | 4a | 2b | 0 | rectum |
| SHCRC-T-22 | Male | 81 | 4a | 2b | 1 | rectum |
| SHCRC-T-23 | Male | 76 | 3 | 2a | 0 | rectum |
| SHCRC-T-24 | Female | 48 | 3 | 0 | 0 | rectum |
| SHCRC-T-25 | Male | 59 | 3 | 0 | 0 | Rectum |
| SHCRC-T-26 | Male | 60 | 4a | 1b | 0 | Rectum |
| SHCRC-T-27 | Female | 41 | 4a | 2b | 0 | colon |
| SHCRC-T-28 | Female | 74 | 4b | 0 | 0 | colon |
| SHCRC-T-29 | Male | 72 | 4a | 1a | 0 | Rectum |
| SHCRC-T-30 | Male | 56 | 3 | 0 | 0 | Rectum |
| SHCRC-T-31 | Male | 53 | 3 | 2b | 0 | colon |
| SHCRC-T-32 | Male | 60 | 3 | 1a | 0 | Rectum |
| SHCRC-T-33 | Male | 58 | 2 | 1a | 0 | Rectum |
| SHCRC-T-34 | Female | 72 | 2 | 2a | 0 | Rectum |
| SHCRC-T-35 | Female | 61 | 3 | 2a | 0 | Rectum |
| SHCRC-T-36 | Male | 60 | 3 | 2b | 0 | Rectum |
| SHCRC-T-37 | Male | 49 | 2 | 0 | 0 | colon |
| SHCRC-T-38 | Female | 66 | 2 | 0 | 0 | Rectum |
| SHCRC-T-39 | Female | 85 | 3 | 2a | 0 | Rectum |
| SHCRC-T-40 | Female | 49 | 3 | 0 | 0 | Rectum |
| SHCRC-T-41 | Female | 61 | 2 | 0 | 0 | Rectum |
| SHCRC-T-42 | Male | 65 | 3 | 0 | 0 | Rectum |
| SHCRC-T-43 | Male | 79 | 4a | 0 | 0 | colon |
| SHCRC-T-44 | Female | 68 | 2 | 0 | 0 | Rectum |
| SHCRC-T-45 | Male | 62 | 4a | 0 | 0 | Rectum |
| SHCRC-T-46 | Male | 48 | 3 | 1b | 0 | Rectum |
| SHCRC-T-47 | Male | 72 | 2 | 1b | 0 | Rectum |
| SHCRC-T-48 | Female | 39 | 4a | 1b | 0 | Rectum |
| SHCRC-T-49 | Female | 61 | 3 | 0 | 0 | Rectum |
| SHCRC-T-50 | F | 65 | 4a | 2a | 0 | Rectum |
| SHCRC-T-51 | Female | 78 | 2 | 1b | 0 | Rectum |
| SHCRC-T-52 | Female | 62 | 4b | 0 | 0 | Rectum |
| SHCRC-T-53 | Female | 59 | 4b | 0 | 0 | colon |
| SHCRC-T-54 | M | 45 | 4a | 1b | 0 | colon |
| SHCRC-T-55 | Male | 61 | 3 | 0 | 0 | Rectum |
| SHCRC-T-56 | Male | 67 | 3 | 0 | 0 | Rectum |
| SHCRC-T-57 | Female | 72 | 4a | 0 | 0 | colon |
| SHCRC-T-58 | Female | 62 | 4a | 1b | 0 | colon |
| SHCRC-T-59 | Male | 78 | 4a | 2a | 0 | colon |
| SHCRC-T-60 | Female | 48 | 4a | 0 | 0 | Rectum |
| SHCRC-T-61 | Male | 69 | 3 | 0 | 1a | Rectum |
| SHCRC-T-62 | M | 57 | 4b | 2b | 0 | colon |
| SHCRC-T-63 | Female | 72 | 3 | 1b | 0 | Rectum |
| SHCRC-T-64 | Male | 72 | 4b | 0 | 0 | colon |
| SHCRC-T-65 | Female | 58 | 4a | 1b | 0 | Rectum |
| SHCRC-T-66 | M | 54 | 4a | 1b | 0 | colon |
| SHCRC-T-67 | M | 61 | 4a | 0 | 0 | Rectum |
| SHCRC-T-68 | Male | 73 | 4a | 1b | 0 | Rectum |
| SHCRC-T-69 | Female | 59 | 4a | 1b | 0 | colon |
| SHCRC-T-70 | Male | 58 | 4b | 0 | 0 | Rectum |
| SHCRC-T-71 | Male | 71 | 4a | 1b | 0 | colon |
| SHCRC-T-72 | Male | 61 | 4a | 0 | 0 | Rectum |
| SHCRC-T-73 | Male | 38 | 4a | 2b | 0 | Rectum |
| SHCRC-T-74 | Male | 67 | 4a | 0 | 0 | colon |
| SHCRC-T-75 | Female | 64 | 3 | 0 | 0 | colon |
| SHCRC-T-76 | Male | 65 | 4a | 0 | 0 | colon |
| SHCRC-T-77 | Female | 63 | 3 | 0 | 0 | Rectum |
| SHCRC-T-78 | Male | 52 | 4b | 2a | 0 | Rectum |
| SHCRC-T-79 | Male | 33 | 3 | 0 | 0 | Rectum |
| SHCRC-T-80 | Male | 61 | 3 | 2a | 0 | colon |
| SHCRC-T-81 | M | 74 | 3 | 0 | 0 | Rectum |
| SHCRC-T-82 | F | 62 | 3 | 2b | 0 | Rectum |
| SHCRC-T-83 | M | 85 | 4a | 0 | 0 | Rectum |
| SHCRC-T-84 | M | 74 | 2 | 0 | 0 | Rectum |
| SHCRC-T-85 | M | 53 | 3 | 0 | 0 | Rectum |
| SHCRC-T-86 | M | 64 | 4a | 1a | 0 | colon |
| SHCRC-T-87 | F | 68 | 4a | 2b | 1a | colon |
| SHCRC-T-88 | M | 37 | 4a | 1b | 0 | Rectum |
| SHCRC-T-89 | M | 61 | 4b | 2a | 0 | Rectum |
| SHCRC-T-90 | F | 70 | 3 | 2a | 0 | colon |
| SHCRC-T-91 | Male | 77 | 4a | 1b | 0 | Rectum |
| SHCRC-T-92 | Female | 65 | 3 | 2b | 0 | Rectum |
| SHCRC-T-93 | Male | 51 | 4a | 2a | 0 | Rectum |
| SHCRC-T-94 | Male | 77 | 4a | 1b | 0 | Rectum |
| SHCRC-T-95 | Female | 90 | 4a | 0 | 0 | Rectum |
| SHCRC-T-96 | Female | 82 | 3 | 0 | 0 | Rectum |
| SHCRC-T-97 | Male | 74 | 3 | 0 | 0 | Rectum |
| SHCRC-T-98 | Female | 66 | 4a | 2a | 0 | Rectum |
| SHCRC-T-99 | Female | 79 | 2 | 0 | 0 | Rectum |
| SHCRC-T-100 | Male | 75 | 3 | 1b | 0 | colon |
| SHCRC-T-101 | Female | 67 | 2 | 0 | 0 | Rectum |
| SHCRC-T-102 | M | 74 | 2 | 2a | 0 | colon |
| SHCRC-T-103 | F | 78 | 4a | 0 | 0 | colon |
| SHCRC-T-104 | Female | 77 | 3 | 0 | 0 | Rectum |
| SHCRC-T-105 | Male | 45 | 4a | 0 | 0 | Rectum |
| SHCRC-T-106 | Male | 83 | 4b | 2b | 0 | colon |
| SHCRC-T-107 | Female | 77 | 2 | 0 | 0 | Rectum |
| SHCRC-T-108 | Male | 61 | 4a | 0 | 0 | colon |
| SHCRC-T-109 | Male | 69 | 4a | 0 | 0 | colon |
