# Supplementary material for: Long non‐coding RNA ADAMTS9‐AS1 suppresses colorectal cancer by inhibiting the Wnt/β‐catenin signalling pathway and is a potential diagnostic biomarker
Source: J Cell Mol Med. 2020 Sep 5;24(19):11318–29. doi: 10.1111/jcmm.15713 (PMC7576284; doi:10.1111/jcmm.15713)
Supplement: Supplementary file 6 — Table S3 [file JCMM-24-11318-s006.docx]

**Table S3. Demographic and clinical characteristics of individuals with CRC who**

**provide serums**

| **Sample ID** | **Gende** | **Age** | **T** | **N** | **M** | **Tumor** |
| --- | --- | --- | --- | --- | --- | --- |
|  |  | **Year** | **Stage** | **Stage** | **Stage** | **Location** |
| SHCRC-P-1 | Male | 84 | 3 | 2b | M0 | Rectum |
| SHCRC-P-2 | Male | 57 | 3 | 1b | M0 | Rectum |
| SHCRC-P-3 | Male | 63 | 3 | 1b | M0 | Rectum |
| SHCRC-P-4 | Male | 55 | 3 | 0 | M0 | Colon |
| SHCRC-P-5 | Male | 84 | 3 | 0 | M0 | Colon |
| SHCRC-P-6 | Female | 47 | 3 | 0 | M0 | Colon |
| SHCRC-P-7 | Female | 58 | 2 | 0 | M0 | Rectum |
| SHCRC-P-8 | Male | 78 | 3 | 0 | M0 | Rectum |
| SHCRC-P-9 | Female | 84 | 4a | 0 | M0 | Colon |
| SHCRC-P-10 | Female | 42 | 2 | 1b | M0 | Rectum |
| SHCRC-P-11 | Male | 70 | 3 | 2a | M0 | Colon |
| SHCRC-P-12 | Female | 45 | 3 | 1b | M0 | Colon |
| SHCRC-P-13 | Male | 50 | 3 | 1b | M0 | Rectum |
| SHCRC-P-14 | Female | 70 | Tis | 0 | M0 | Rectum |
| SHCRC-P-15 | Male | 41 | 3 | 2b | M0 | Rectum |
| SHCRC-P-16 | Female | 66 | 3 | 1 | M0 | Colon |
| SHCRC-P-17 | Male | 78 | 3 | 1a | M0 | Rectum |
| SHCRC-P-18 | Female | 55 | 3 | 2a | M0 | Rectum |
| SHCRC-P-19 | Female | 41 | 3 | 0 | M0 | Colon |
| SHCRC-P-20 | Male | 68 | 0 | 0 | M0 | Colorectum |
| SHCRC-P-21 | Female | 62 | 3 | 0 | M0 | Colorectum |
| SHCRC-P-22 | Male | 69 | 2 | 0 | M0 | Rectum |
| SHCRC-P-23 | Male | 78 | 3 | 2a | M0 | Rectum |
| SHCRC-P-24 | Male | 41 | 3 | 2a | M0 | Colon |
| SHCRC-P-25 | Male | 75 | 1 | 0 | M0 | Rectum |
| SHCRC-P-26 | Male | 35 | 3 | 0 | M0 | Colon |
| SHCRC-P-27 | Female | 45 | 2 | 0 | M0 | Rectum |
| SHCRC-P-28 | Female | 54 | 2 | 0 | M0 | Colon |
| SHCRC-P-29 | Female | 61 | 4a | 1b | M0 | Rectum |
| SHCRC-P-30 | Male | 68 | 3 | 0 | M0 | Rectum |
| SHCRC-P-31 | Female | 54 | 2 | 0 | M0 | Colon |
| SHCRC-P-32 | Female | 41 | 3 | 0 | M0 | Rectum |
| SHCRC-P-33 | Male | 59 | 3 | 1a | M0 | Rectum |
| SHCRC-P-34 | Male | 82 | 4a | 1a | M0 | Rectum |
| SHCRC-P-35 | Male | 75 | 3 | 0 | M0 | Rectum |
| SHCRC-P-36 | Male | 55 | 3 | 2a | M0 | Colon |
| SHCRC-P-37 | Female | 66 | 2 | 0 | M0 | Rectum |
| SHCRC-P-38 | Female | 73 | Tx | Nx | M1 | Rectum |
| SHCRC-P-39 | Male | 61 | 3 | 1a | M0 | Rectum |
| SHCRC-P-40 | Female | 61 | 3 | 1a | M0 | Colon |
| SHCRC-P-41 | Male | 62 | 3 | 1a | M0 | Colon |
| SHCRC-P-42 | Female | 36 | 3 | 2a | M0 | Colon |
| SHCRC-P-43 | Male | 68 | 3 | 2a | M1 | Colon |
| SHCRC-P-44 | Female | 61 | 3 | 0 | M0 | Rectum |
| SHCRC-P-45 | Male | 51 | 1 | 0 | M0 | Colon |
| SHCRC-P-46 | Male | 63 | 2 | 1a | M0 | Rectum |
| SHCRC-P-47 | Male | 62 | 3 | 0 | M1 | Rectum |
| SHCRC-P-48 | Male | 48 | 3 | 0 | M0 | Colon |
| SHCRC-P-49 | Female | 84 | 3 | 2a | M0 | Colon |
| SHCRC-P-50 | Female | 62 | 2 | 0 | M0 | Rectum |
| SHCRC-P-51 | Male | 66 | 3 | 2a | M0 | Colon |
| SHCRC-P-52 | Female | 58 | 2 | 0 | M0 | Rectum |
| SHCRC-P-53 | Male | 60 | 4a | 1a | M0 | Rectum |
| SHCRC-P-54 | Male | 62 | 3 | 2a | M0 | Rectum |
| SHCRC-P-55 | Female | 88 | 3 | 1a | M0 | Colon |
| SHCRC-P-56 | Male | 66 | 4a | 1a | M0 | Rectum |
| SHCRC-P-57 | Male | 52 | 3 | 0 | M0 | Rectum |
| SHCRC-P-58 | Male | 34 | 4a | 0 | M0 | Colon |
| SHCRC-P-59 | Female | 70 | 4a | 0 | M0 | Rectum |
| SHCRC-P-60 | Female | 73 | 4a | 1a | M0 | Rectum |
| SHCRC-P-61 | Male | 24 | 3 | 0 | M0 | Rectum |
| SHCRC-P-62 | Female | 54 | 3 | 1a | M0 | Colon |
| SHCRC-P-63 | Male | 59 | 4a | 2a | M0 | Rectum |
| SHCRC-P-64 | Female | 76 | 2 | 0 | M0 | Rectum |
| SHCRC-P-65 | Female | 60 | 3 | 1a | M0 | Rectum |
| SHCRC-P-66 | Female | 54 | 4a | 1a | M0 | Rectum |
| SHCRC-P-67 | Male | 65 | 3 | 0 | M0 | Colon |
| SHCRC-P-68 | Male | 80 | 3 | 0 | M0 | Colon |
| SHCRC-P-69 | Male | 59 | 3 | 2a | M0 | Colon |
| SHCRC-P-70 | Female | 66 | 3 | 2a | M0 | Colon |
| SHCRC-P-71 | Male | 53 | 4a | 0 | M0 | Rectum |
| SHCRC-P-72 | Male | 28 | 3 | 2b | M0 | Rectum |
| SHCRC-P-73 | Male | 41 | 3 | 0 | M0 | Colon |
| SHCRC-P-74 | Male | 67 | 3 | 2b | M0 | Rectum |
| SHCRC-P-75 | Male | 49 | 3 | 0 | M0 | Rectum |
| SHCRC-P-76 | Female | 59 | 4a | 2b | M0 | Colon |
| SHCRC-P-77 | Male | 52 | x | 1a | M0 | Rectum |
| SHCRC-P-78 | Male | 45 | 4a | 1a | M0 | Rectum |
| SHCRC-P-79 | Female | 78 | 4a | 0 | M0 | Rectum |
| SHCRC-P-80 | Male | 63 | 4a | 0 | M0 | Rectum |
| SHCRC-P-81 | Female | 58 | 4a | 0 | M0 | Rectum |
| SHCRC-P-82 | Male | 46 | 3 | 0 | M0 | Colon |
| SHCRC-P-83 | Male | 69 | 3 | 1a | M0 | Colon |
| SHCRC-P-84 | Male | 78 | 4a | 1a | M0 | Rectum |
| SHCRC-P-85 | Female | 71 | 4a | 0 | M0 | Rectum |
| SHCRC-P-86 | Male | 77 | 3 | 2b | M1 | Colon |
| SHCRC-P-87 | Female | 69 | 3 | 1a | M1 | Colon |
| SHCRC-P-88 | Male | 54 | 4a | 1a | M0 | Rectum |
| SHCRC-P-89 | Female | 69 | 3 | 1a | M0 | Rectum |
| SHCRC-P-90 | Male | 70 | 4a | 0 | M0 | Rectum |
| SHCRC-P-91 | Female | 74 | 3 | 2b | M0 | Colon |
| SHCRC-P-92 | Male | 48 | 4a | 0 | M0 | Colon |
| SHCRC-P-93 | Male | 56 | is | 0 | M0 | Rectum |
| SHCRC-P-94 | Male | 70 | 3 | 0 | M0 | Colon |
| SHCRC-P-95 | Male | 66 | 4a | 0 | M0 | Rectum |
| SHCRC-P-96 | Female | 76 | 3 | 0 | M0 | Rectum |
| SHCRC-P-97 | Female | 60 | 4a | 1a | M0 | Rectum |
| SHCRC-P-98 | Male | 63 | 3 | 1a | M0 | Rectum |
| SHCRC-P-99 | Male | 60 | 4a | 0 | M0 | Rectum |
| SHCRC-P-100 | Male | 58 | 3 | 1a | M0 | Rectum |
| SHCRC-P-101 | Male | 59 | 3 | 0 | M0 | Colon |
| SHCRC-P-102 | Male | 66 | 4a | 0 | M0 | Rectum |
| SHCRC-P-103 | Male | 68 | 4a | 0 | M0 | Rectum |
| SHCRC-P-104 | Male | 54 | 3 | 1a | M0 | Rectum |
| SHCRC-P-105 | Male | 73 | 3 | 0 | M0 | Colorectum |
| SHCRC-P-106 | Female | 61 | 2 | 0 | M0 | Rectum |
| SHCRC-P-107 | Male | 74 | 3 | 1a | M0 | Colon |
| SHCRC-P-108 | Female | 62 | 3 | 0 | M0 | Colorectum |
| SHCRC-P-109 | Male | 45 | 3 | 0 | M0 | Colon |
| SHCRC-P-110 | Male | 48 | 4a | 1a | M0 | Rectum。 |
| SHCRC-P-111 | Female | 71 | 4a | 1a | M0 | Rectum |
| SHCRC-P-112 | Female | 53 | 2 | 0 | M0 | Rectum |
| SHCRC-P-113 | Female | 78 | 4a | 2b | M0 | Rectum |
| SHCRC-P-114 | Female | 60 | 2 | 0 | M0 | Rectum |
| SHCRC-P-115 | Male | 67 | 2 | 0 | M0 | Rectum |
| SHCRC-P-116 | Female | 73 | 4a | 0 | M0 | Rectum |
| SHCRC-P-117 | Female | 69 | 1 | 0 | M0 | Colon |
| SHCRC-P-118 | Male | 70 | 3 | 2a | M0 | Rectum |
| SHCRC-P-119 | Male | 66 | 2 | 0 | M0 | Rectum |
| SHCRC-P-120 | Female | 73 | 4a | 0 | M0 | Rectum |
| SHCRC-P-121 | Female | 54 | 3 | 1a | M0 | Colon |
| SHCRC-P-122 | Female | 50 | 2 | 1a | M0 | Colon |
| SHCRC-P-123 | Female | 78 | 3 | 2a | M0 | Colon |
| SHCRC-P-124 | Male | 50 | 2 | 0 | M0 | Rectum |
| SHCRC-P-125 | Male | 66 | 4a | 2b | M0 | Rectum |
| SHCRC-P-126 | Female | 65 | 3 | 2b | M1 | Colon |
| SHCRC-P-127 | Female | 71 | 3 | 1a | M0 | Colorectum |
| SHCRC-P-128 | Male | 68 | 3 | 0 | M0 | Colon |
| SHCRC-P-129 | Female | 77 | Tis | 0 | M0 | Rectum |
| SHCRC-P-130 | Female | 61 | 3 | 1a | M0 | Colon |
